# Supplementary material for: Causal Relationship between Aging and Anorexia Nervosa: A White-Matter-Microstructure-Mediated Mendelian Randomization Analysis
Source: Biomedicines. 2024 Aug 16;12(8):1874. doi: 10.3390/biomedicines12081874 (PMC11351342; doi:10.3390/biomedicines12081874)
Supplement: Supplementary file 1 [file biomedicines-12-01874-s001.zip › Supplementary Figure/Figure S4/Singe SNP plot of MR analysis for TL to ubm-b-1582.pdf]

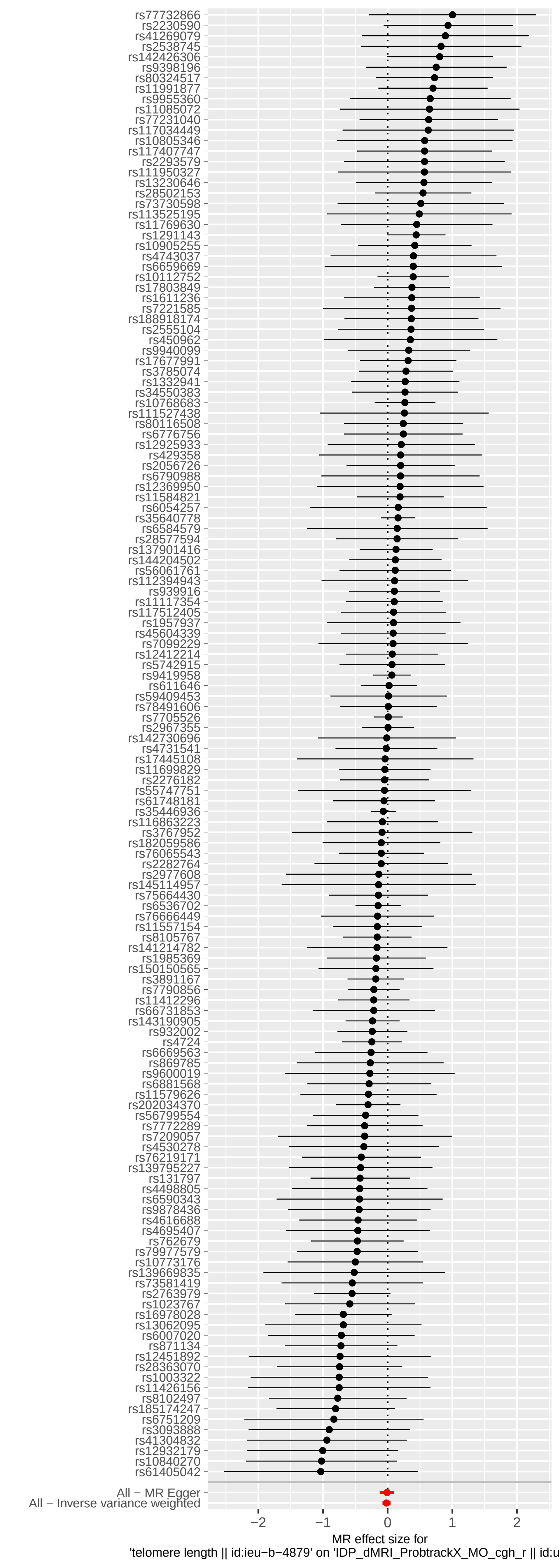
rs77732866
rs2230590
rs41269079
rs2538745
rs142426306
rs9398196
rs80324517
rs11991877
rs9955360
rs11085072
rs77231040
rs117034449
rs10805346
rs117407747
rs2293579
rs111950327
rs13230646
rs28502153
rs73730598
rs113525195
rs11769630
rs1291143
rs10905255
rs4743037
rs6659669
rs10112752
rs17803849
rs1611236
rs7221585
rs188918174
rs2555104
rs450962
rs9940099
rs17677991
rs3785074
rs1332941
rs34550383
rs10768683
rs111527438
rs80116508
rs6776756
rs12925933
rs429358
rs2056726
rs6790988
rs12369950
rs11584821
rs6054257
rs35640778
rs6584579
rs28577594
rs137901416
rs144204502
rs56061761
rs112394943
rs939916
rs11117354
rs117512405
rs1957937
rs45604339
rs7099229
rs12412214
rs5742915
rs9419958
rs611646
rs59409453
rs78491606
rs7705526
rs2967355
rs142730696
rs4731541
rs17445108
rs11699829
rs2276182
rs55747751
rs61748181
rs35446936
rs116863223
rs3767952
rs182059586
rs76065543
rs2282764
rs2977608
rs145114957
rs75664430
rs6536702
rs76666449
rs11557154
rs8105767
rs141214782
rs1985369
rs150150565
rs3891167
rs7790856
rs11412296
rs66731853
rs143190905
rs932002
rs4724
rs6669563
rs869785
rs9600019
rs6881568
rs11579626
rs202034370
rs56799554
rs7772289
rs7209057
rs4530278
rs76219171
rs139795227
rs131797
rs4498805
rs6590343
rs9878436
rs4616688
rs4695407
rs762679
rs79977579
rs10773176
rs139669835
rs73581419
rs2763979
rs1023767
rs16978028
rs13062095
rs6007020
rs871134
rs12451892
rs28363070
rs1003322
rs11426156
rs8102497
rs185174247
rs6751209
rs3093888
rs41304832
rs12932179
rs10840270
rs61405042
All − MR Egger
All − Inverse variance weighted
−2
−1
0
1
2
MR effect size for
'telomere length || id:ieu−b−4879' on 'IDP_dMRI_ProbtrackX_MO_cgh_r || id:u
